# The Patient Selection Criteria for Veno-arterial Extracorporeal Mechanical Oxygenation

**DOI:** 10.7759/cureus.5709

**Published:** 2019-09-20

**Authors:** Sandeep Banga, Abhiram Challa, Avani R Patel, Shantanu Singh, Vamsi K Emani

**Affiliations:** 1 Cardiology, West Virginia University School of Medicine, Morgantown, USA; 2 Internal Medicine, West Virginia University School of Medicine, Morgantown, USA; 3 Internal Medicine, Northern California Kaiser Permanente, Fremont, USA; 4 Pulmonary Medicine, Marshall University School of Medicine, Huntington, USA; 5 Internal Medicine, University of Illinois College of Medicine at Peoria, Order of St. Francis Medical Centre, Peoria, USA

**Keywords:** extracorporeal membrane oxygenation (ecmo), venoarterial extracorporeal membrane oxygenation (va-ecmo), cardiac arrest, cardiogenic shock

## Abstract

Veno-arterial extracorporeal membrane oxygenation (VA-ECMO) plays a crucial role in the management of patients with refractory cardiac and pulmonary dysfunction by providing temporary mechanical hemodynamic and respiratory support. However, the paucity of guidelines on indications for administering it and the failure to timely initiate VA-ECMO often result in a high in-hospital mortality rate and poor six-month outcomes after VA-ECMO deployment. Due to ethical issues, randomized controlled studies with VA-ECMO have not been conducted so that no recommended evidence-based guidelines exist for VA-ECMO patient-selection criteria. Therefore, the indication for administering the device depends solely on expert opinion after reviewing the literature. We conducted a review of the current literature to better understand and classify the need for proper patient selection, including proven indications for VA-ECMO.

## Introduction and background

Extracorporeal membrane oxygenation (ECMO) is a device designed to provide cardiopulmonary life support by oxygenating drained blood from the vascular system outside the body and filtering it back into the body via a mechanical pump. The device has been used since the 1970s and has completely transformed how physicians view supportive therapy for cardiac patients [1]. There are two types of ECMO: veno-arterial (VA-ECMO) and veno-venous (VV-ECMO). As the name implies, VA-ECMO retrieves de-oxygenated blood from the venous system and reinfuses the oxygenated blood into the arterial system, whereas VV-ECMO retrieves and delivers the blood to the venous system. Hence, both forms can provide respiratory support through the gas exchange; however, only VA-ECMO supports hemodynamic circulation. The most common indications for administering VA-ECMO are cardiogenic shock and cardiac arrest. However, most studies have observed many complications following the use of the device, such as thrombosis and bleeding seen in 22% & 79% of the patients, respectively. Moreover, there are hardly any guidelines on indications for administering the device and timing of its initiation [2]. Therefore, we felt the need to discuss the patient-selection criteria for VA-ECMO and classify the reported relative indications to provide a better understanding to reduce complications and mortality rates.

## Review

Although VA-ECMO is a form of supportive therapy, the appropriate selection of patients with the right type of configuration including site, management, and complication apprehension is necessary to acquire optimal results. According to the data from the annual international Extracorporeal Life Support Organization (ELSO) registry reports through January 2015, VA-ECMO supported 18,700 cardiac cases and 5,100 cases for extracorporeal cardiopulmonary resuscitation (ECPR) [3]. Given the increased adoption of this invasive management technique, knowledge of the appropriate indications for VA-ECMO is necessary to understand both outcome expectations and complication reduction. 

Prior to understanding the indications, it is essential to get a grasp of the hemodynamics of ECMO. Precautions may be required to avoid a fluid overloaded state in the lungs and the left ventricle (LV) prior to ECMO administration. ECMO can unload the right atrium (RA), right ventricle (RV), and the central veins; however, it does not intrinsically unload the LV, especially when the contractile function of the LV is compromised. Increased time on a VA-ECMO with a poorly contractile heart significantly increases the afterload, which can ultimately cause increased LV end-diastolic pressure (LVEDP) and wall tension. This results in increased myocardial oxygen consumption and makes the patient susceptible to ischemia-mediated necrosis. Therefore, it is not an ideal support for isolated LV dysfunction and anticipating measures to unload the LV prior to ECMO administration is necessary [4]. These methods include inotropes, vasodilator, intra-aortic balloon pumps (IABP), balloon atrial septostomy, surgical LV vent, percutaneous LV vent, percutaneous ventricular support, and an off-pump central VA-ECMO [4].

**Figure 1:**
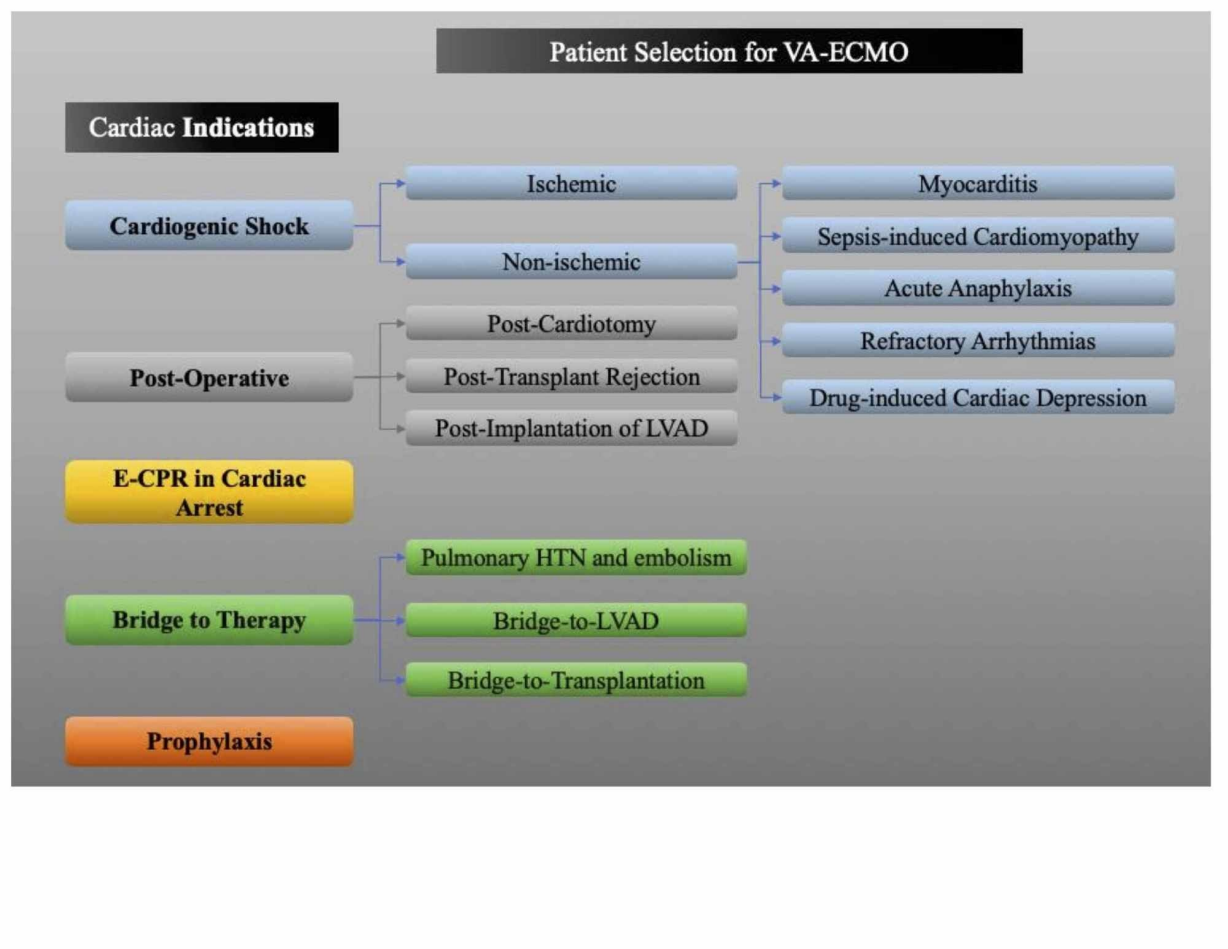
Patient selection for VA-ECMO therapy. This is a created diagram regarding patient selection for VA-ECMO based on cardiac indication. VA-ECMO: veno-arterial extracorporeal membrane oxygenation; E-CPR: extracorporeal cardiopulmonary resuscitation; LVAD: left ventricular assist device.

Adult patients with the following conditions (Figure 1) have experienced the maximum benefit from the VA-ECMO therapy, demonstrating the importance of adopting appropriate patient-selection criteria for VA-ECMO support. 

Cardiogenic shock 

Until several years ago, IABP were frequently preferred in the management of cardiogenic shock. However several randomized studies have demonstrated IABP support to be ineffective [5]. Although the adoption of VA-ECMO has been on the rise, data from large studies are limited, and most of the existing data originate from randomized and registry studies. Of the available devices [ECMO, Impella (Abiomed, Danvers, MA), TandemHeart (CardiacAssist, Pittsburgh, PA)], ECMO application in cardiogenic shock has proved to be easier and faster, making it the favored therapy among doctors [5].

VA-ECMO in cardiogenic shock is considered after all other relatively less-invasive measures have been attempted. These include volume administration, high-dose vasoconstrictor support, IABP, and mechanical ventilation (if appropriate). If cardiogenic shock persists despite these measures, the common indications for the application of VA-ECMO are as follows: low cardiac output (cardiac index of <2 l/m/m^2^); lactate levels of >50 mg/dL; central venous O2 saturation of <65%; and hypotension (systolic blood pressure of <90 mm Hg) [6].

Cardiogenic shock can be classified into either ischemic (acute coronary syndrome) or non-ischemic in origin based on its precipitating factor.

Acute Coronary Syndrome

Acute causes such as a myocardial infarction (MI) or worsening of chronic ischemic processes can contribute to ischemia-induced cardiogenic shock. Non-randomized studies reveal that using VA-ECMO in conjunction with revascularization enhances survival rates and lowers the six-month mortality rates in MI-induced cardiogenic shocks [7]. However, cautious use is recommended in patients of advanced age and comorbidities as a higher risk of complications are noticed. Previously published studies have demonstrated the beneficial effects of VA-ECMO in patients with acute coronary syndrome (Table 1).

**Table 1:** Research studies supporting the use of VA-ECMO in acute coronary syndrome. VA-ECMO: venoarterial extracorporeal membrane oxygenation.

Study	Determined conclusion
Sheu et al. (2010)	The early initiation of VA-ECMO in cardiogenic shock patients caused by myocardial infarction was found to have improved 30-day outcomes post therapy in patients [8].
Tsao et al. (2012)	Patients of cardiogenic shock secondary to acute myocardial infarction were treated with VA-ECMO, which led to improved 30-day and one-year outcomes in patients [9].

Cardiogenic Shock of Non-Ischemic Origin

Fulminant myocarditis and sepsis-induced cardiomyopathy are the most common causes of non-ischemic cardiogenic shock, and VA-ECMO is frequently used in their management [2]. In myocarditis-induced cardiogenic shock, VA-ECMO serves as a bridge to therapy, demonstrating comparable outcomes to that of myocarditis without shock. VA-ECMO is considered a bridge therapy in sepsis-induced cardiomyopathy only when the benefits outweigh the risks of thrombosis and bleeding; however, there is a lack of adequate data to confirm this. Other causes of non-ischemic cardiogenic shock include refractory cardiac arrhythmic storms, acute anaphylaxis, medication-induced cardiac depression, and severe hypothermia with cardiac instability [2]. Previously published studies have shown the beneficial effects of VA-ECMO for patients with cardiogenic shock of non-ischemic origin (Table 2).

**Table 2:** Research studies supporting the use of VA-ECMO in cardiogenic shock of non-ischemic origin. VA-ECMO: venoarterial extracorporeal membrane oxygenation; ECMO: extracorporeal membrane oxygenation.

Study	Determined conclusion
Asaumi et al. (2005)	Fulminant myocarditis treated with ECMO was proven to have a good clinical outcome that is comparable to outcomes seen in patients of non-fulminant myocarditis [10].
Bréchot et al. (2013)	VA-ECMO therapy rescued more than 70% of patients who developed refractory cardiovascular dysfunction caused by severe bacterial septic shock [11].

Postoperative patients

The use of VA-ECMO in postoperative cases is usually done for patients who are post-transplant, those who have undergone implantation of ventricular assist device (VAD), and those who have had cardiotomy. In the earlier two types, VA-ECMO has had a beneficial impact on patient survival (Table 3).

**Table 3:** Research studies supporting the use of VA-ECMO in post-transplant and post-implantation of VAD. VA-ECMO: venoarterial extracorporeal membrane oxygenation; ECMO: extracorporeal membrane oxygenation; VAD: ventricular assist device.

Study	Determined conclusion
Marasco et al. (2010)	ECMO-supported patients showed a good survival-to-discharge rate in primary graft failure after heart transplantation [12].
Barth et al. (2012)	ECMO was determined to be beneficial for supporting patients awaiting high-urgency heart transplantation and as a short-term bridge to surgery [13].
Lebreton et al. (2011)	Patients with recently implanted VADs benefited from ECMO-supported therapy and demonstrated improved patient prognosis [14].
Takayama et al. (2012)	Patients with newly developed percutaneous right-VAD were treated with ECMO and demonstrated hemodynamic improvement [15].

The use of VA-ECMO is beneficial in post-transplant cases with primary graft failure after heart or heart-lung transplantation. Mortality rates after primary graft rejection are substantial. However, if the patient survives the initial event, deploying VA-ECMO has resulted in comparable outcomes to those without primary graft rejection [2]. Prompt initiation of VA-ECMO serves as the most critical factor in determining outcomes.

In cases of right heart failure, post-left ventricular assist device (LVAD) implantation benefits by allowing the RV to adapt to the hemodynamic changes by providing cardiac support. A VAD is an implantable mechanical pump that helps to pump blood from the ventricles to the rest of the body.

The use of VA-ECMO in postcardiotomy patients is limited to those who are unable to wean immediately from cardiopulmonary bypass. However, with a survival rate of 30%, the postcardiotomy use of this invasive technique is deemed quite unsatisfactory [7]. A previous study performed with 517 patients found that ECMO therapy given to post-cardiotomy cardiogenic shock patients led to a high morbidity and high mortality rate [16]. The absence of prior cardiac disease or comorbidities along with a temporary cessation of the cardiopulmonary system has exhibited more beneficial outcomes [4].

Bridge-to-therapy

VA-ECMO may function as a bridge to recovery when the cardiac injury is potentially reversible; yet it is generally favored as a bridge-to-destination therapy, such as in LVAD placement and cardiac transplantation. Previously published studies have determined VA-ECMO to have a beneficial effect as a bridge-to-recovery therapy in patients with pulmonary hypertension and pulmonary embolism (see Table 4).

**Table 4:** Research studies supporting the use of VA-ECMO in bridge-to-therapy, pulmonary embolism, and pulmonary hypertension patients. VA-ECMO: venoarterial extracorporeal membrane oxygenation; ECMO: extracorporeal membrane oxygenation; PAH: pulmonary arterial hypertension.

Study	Determined conclusion
Fitzpatrick et al. (2009)	Patient treated earlier with a biventricular assist device had dramatic improvement in survival [17].
Maggio et al. (2007)	The use of ECMO in massive pulmonary embolism patients was proven to increase overall survival rates up to 62% when combined with anticoagulation and surgical embolectomy [18].
Munakata et al. (2012)	The use of ECMO in massive pulmonary embolism patients improved prognosis in the 30-day survival rate of patients (70%) [19].
Strueber et al. (2009)	Four patients with cardiogenic shock due to end‐stage pulmonary hypertension were successfully treated with VA-ECMO and bridged to bilateral lung transplantation and combined heart–lung transplantation [20].
Abrams et al. (2013)	VA-ECMO is a feasible treatment modality for decompensated PAH, leading to significant improvement in hemodynamics, gas exchange, and end-organ perfusion [21].

A recent study shows that a left ventricular end-diastolic volume (LVEDV) of <54 mm, diminished hepatic function, and RV failure correlate with unsatisfactory outcomes in patients requiring VA-ECMO as a bridge therapy toward LVAD implantation. However, assessing for post-LVAD RV failure is a major challenge in LVAD-implant-bridged VA-ECMO patients. This is due to the loading of the RV by the VA-ECMO, which creates difficulty in performing an accurate hemodynamic evaluation of the RV function. Although RV failure produces poor outcomes, the early administration of a biventricular assist device was reported to improve outcomes [17]. Hence, preoperative identification for RV failure to determine the need for a biventricular assist device can ultimately prevent end-organ failure.

Additionally, in cases of pulmonary hypertension and embolism, VA-ECMO operates as a bridge to therapeutic procedures, such as thrombectomy, particularly when decompensation occurs early. 

Extracorporeal cardiopulmonary resuscitation in cardiac arrest

E-CPR is defined as the reestablishment of circulation in cardiac arrest with the aid of extracorporeal supportive devices such as VA-ECMO. A review of the existing retrospective studies has been recently published elsewhere [22]. The literature proves that VA-ECMO provides satisfactory systemic circulation in refractory cardiac arrest [5]. Data suggest that VA-ECMO enhances in-hospital survival and survival without neurological impairment up to two years when applied in properly selected patients alongside cardiopulmonary resuscitation (CPR) [23-24]. Although a set of recommended inclusion criteria for VA-ECMO in refractory cardiac arrest exist, they only provide a framework for decisions. They may be adjusted for individual patients. All of the following inclusion criteria are required to be met for VA-ECMO deployment: cardiac arrest is witnessed in the patient; the patient has received bystander CPR; the patient is of <75 years old; no return of spontaneous circulation (ROSC) is observed in the patient after 10 minutes of professional CPR.

Despite the inclusion criteria requiring the age to be less than 75 years, the age limit depends chiefly on comorbidities and biological age. Furthermore, recent reports suggest that E-CPR may attain reasonable outcomes in elderly patients also [25-27]. Thus, age may not invalidate patients for E-CPR. The proposed exclusion criteria are as follows (one criterion is sufficient) [28]: severe comorbidity (cancer, end-stage liver cirrhosis, etc.); pre-existing brain damage/cognitive impairment; the interval between the patient receiving CPR and starting of clinical treatment exceeding an hour (could be extended in cases of young patients who have received optimal CPR needing time for transfer). Several additional relative contraindications include a baseline pH of <6.8 and a baseline lactate level of >15 mmol/L. The only exception to the criteria is accidental hypothermia.

Prophylaxis

VA-ECMO can be deliberated in the prophylactic management of high-risk percutaneous cardiac interventions [2]. Although it is a developing indication, when a severely complicated course is expected, timely pre- and peri-procedural use of this device may be considered.

Risk assessment 

Several risk scores were created for calculating the odds of survival till hospital discharge for VA-ECMO, namely survival after VA-ECMO (SAVE) and simple cardiac ECMO scores. However, these scores are modestly discriminating at best. The SAVE score was developed using the data from 3,846 patients registered in the ELSO registry. Using the data, the SAVE score was established to help estimate patient survival [29]. This was done by assessing five risk categories: the diagnosis, age, weight, pre-ECMO organ failure, and duration of intubation pre-ECMO. 

## Conclusions

VA-ECMO is a highly effective therapy for patients with cardiogenic shock and cardiac arrest. It is also favored as a bridge therapy as various studies have shown that VA-ECMO can have a beneficial effect as a bridge-to-recovery therapy in patients with pulmonary hypertension and pulmonary embolism. VA-ECMO also has improved outcomes for post-cardiac surgery patients. To further explore the possibilities of VA-ECMO utilization, larger randomized and non-randomized clinical trials are needed.
